# Prevalence of Hemorrhoids and Their Risk Factors Among the Adult Population in Jazan, Saudi Arabia

**DOI:** 10.7759/cureus.45919

**Published:** 2023-09-25

**Authors:** Imtenan A Oberi, Yazan Omar, Aseel J Alfaifi, Raum A Ayoub, Yara Ajeebi, Sarah H Moafa, Abdelkhalig H Elhilu, Abdu H Ayoub, Ibrahim M Gosadi

**Affiliations:** 1 Faculty of Medicine, Jazan University, Jazan, SAU; 2 College of Pharmacy, Jazan University, Jazan, SAU; 3 Department of Surgery, Faculty of Medicine, Jazan University, Jazan, SAU; 4 Department of Surgery, Jazan General Hospital, Jazan, SAU; 5 Department of Family and Community Medicine, Jazan University, Jazan, SAU

**Keywords:** saudi arabia, jazan, patient preference, lifestyle, constipation, hemorrhoids

## Abstract

Background: Hemorrhoids are defined as the symptomatic enlargement and distal displacement of the normal anal cushions. They can be either internal, external, or internoexternal, according to their position in relation to the dentate line. They can cause significant financial burdens and personal discomfort. However, the typical symptoms of hemorrhoids include bleeding, pain, skin irritation, fecal leakage, prolapse, mucus secretion, and developing a thrombosed hernia. Nonetheless, research has shown that individuals with and without hemorrhoids equally report these symptoms. This study aims to investigate the prevalence of hemorrhoids and their risk factors among adult subjects in Jazan, Saudi Arabia.

Methods: This investigation was a cross-sectional study targeting adults diagnosed with hemorrhoids in the Jazan region. Data collection was performed via a self-administered questionnaire to measure sociodemographic data of the participants, lifestyle factors associated with hemorrhoids, clinical presentations of the disease, and Rome IV criteria for diagnosis of functional constipation. The chi-squared test was used to assess the frequency of hemorrhoid symptoms according to the participants' sociodemographic and lifestyle characteristics.

Results: In the current study, which recruited 361 participants, 59% (216 individuals) reported experiencing at least one symptom of hemorrhoids. The majority of the sample were females (60%), Saudis (96%), and living in urban areas (59%) and had a university education (77.3%). The most common risk factors identified were lack of regular physical activity (83%), prolonged sitting during office work (51%), and consuming food with saturated fat (50%). A total of 44% (159 participants) had a family history of hemorrhoids, and 40% reported suffering from constipation three times or more per week. Only 34% (123 participants) had been diagnosed with hemorrhoids by a physician. Interestingly, the majority of participants (57%) preferred using home remedies instead of seeking medical care from a healthcare professional. When assessing factors associated with experiencing a minimum of six hemorrhoid symptoms per month, age, lifestyle factors, family history of hemorrhoids, and defecation practices were found to be significantly correlated with a higher occurrence of symptoms (with a p-value of less than 0.05).

Conclusion: The current study identified a high prevalence of hemorrhoid symptoms and their risk factors among the adult population in the Jazan region of Saudi Arabia. The findings suggest a need to increase the awareness of the public about hemorrhoids risk factors and the importance of seeking healthcare at an early stage of the disease.

## Introduction

Hemorrhoids are defined as the symptomatic enlargement and distal displacement of the regular anal cushions [1]. They can be either internal, external, or internoexternal, according to their position in relation to the dentate line. According to Goligher's classification, they are classified into four grades with grade I meaning non-prolapsing hemorrhoids; grade II: prolapsing hemorrhoids on straining but reduce spontaneously; grade III: prolapsing hemorrhoids requiring manual reduction, and grade IV: non-reducible prolapsing hemorrhoids, which include acutely thrombosed, incarcerated hemorrhoids [2]. They can cause significant financial burdens and personal discomfort. Hemorrhoids are the third most common gastrointestinal diagnosis for outpatient visits in the US, resulting in nearly four million trips to doctor's offices and emergency rooms annually [1]. Although there is no complete consensus among researchers regarding the pathophysiology of hemorrhoids, several theories attempt to explain their underlying causes. These theories suggest that hemorrhoids may be related to diseases affecting the veins in the anorectal vascular cushions, a weakening of the collagen support in the anal canal, or an increase in arterial flow to the vascular plexus [3]. Various factors have been associated with the development of hemorrhoids. These factors include chronic constipation, pregnancy (as the growing uterus exerts pressure on pelvic veins), and prolonged straining [4]. The most widely accepted theory suggests that constipation leads to the chronic exertion of pressure and the formation of hardened stools, resulting in the deterioration of anal canal tissue and distal deflection of the anal cushions [3]. The different epidemiological characteristics of hemorrhoids and constipation, such as gender, age, socioeconomic status, and ethnicity, suggest that there may be doubts about the significance of constipation as a risk factor for hemorrhoids [5]. Hemorrhoids can have several clinical presentations. However, the typical symptoms of internal hemorrhoids include bleeding, pain, skin irritation, fecal leakage, prolapse, and mucus secretion. In contrast, external hemorrhoids are located beneath the dentate line and are covered by squamous epithelium. They are supplied with somatic nerves that can cause pain. Unless they become thrombosed, external hemorrhoids typically do not show any symptoms [6]. For this reason, other diseases such as colorectal cancer, inflammatory bowel diseases, other colitis, diverticular disease, and angiodysplasia should be put in mind when evaluating symptoms attributed to hemorrhoids [7]. With the exception of fiber, there has been a lack of formal research on medical hemorrhoid treatments. However, the effectiveness of fiber in treating hemorrhoids has yielded conflicting results in previous studies. [1]. Evidence considering the prevalence of hemorrhoids is limited. It is difficult to determine the exact number of people who have hemorrhoids, as many individuals prefer to self-treat instead of seeking medical help [5]. However, recent research has indicated that hemorrhoid incidence is increasing. In a study conducted in 2016 on lower gastrointestinal bleeding (LGIB) in the capital city of Saudi Arabia, Riyadh, it was found that hemorrhoids were the most commonly detected colonoscopy finding among patients with LGIB, accounting for 38.5% of cases [8]. According to a study conducted by Murshid, bleeding was identified as the primary symptom of hemorrhoids in Saudi patients during their initial diagnosis [9]. Although hemorrhoids may cause mild rectal bleeding over a long period of time, it can be difficult to confirm that active bleeding is specifically caused by hemorrhoids in all patients. Therefore, it is considered a probable but unconfirmed source of bleeding since most people with hemorrhoids do not experience it. Although hemorrhoids are a prevalent problem in Saudi Arabia, the risk factors contributing to their development are not well understood. Research has yet to be conducted in this area, and understanding these risk factors is important for improving patient treatment and developing effective prevention strategies. The study aims to investigate the prevalence of hemorrhoids and the prevalence of hemorrhoid risk factors among adult subjects in Jazan, southwest of Saudi Arabia.

## Materials and methods

Study context 

This investigation was a cross-sectional study targeting adults diagnosed with hemorrhoids in the Jazan region in the southwest of Saudi Arabia. Data measurement was performed in online settings. Informed consent was taken from the patients before establishment of the data collection process. The study was performed in accordance with the Declaration of Helsinki. Ethical approval to conduct the study was granted by the Jazan Health Ethics Committee (approval number 2285, dated 31/8/2022). 

Data collection tool 

Data collection was performed via a self-administered questionnaire to measure sociodemographic data of the participants, lifestyle factors associated with hemorrhoids, clinical presentation of the disease, Rome IV criteria for the diagnosis of functional constipation [10], and the effect of hemorrhoids on quality of life among the participants [11]. The sociodemographic components of the questionnaire were related to age, gender, education level, marital status and family history of hemorrhoids, and health condition associated with hemorrhoids. Lifestyle components involved asking about smoking history and practices of Khat chewing and chewed tobacco use, work condition, physical activity, and diet behavior. The clinical presentation of the disease was measured by assessing the frequency of hemorrhoid symptoms including the appearance of hemorrhoidal mass, pain, anal itchiness, bleeding, or discharge, and sagging of the hemorrhoids. The Likert scale was used to assess the frequency of these symptoms by asking whether these symptoms occur monthly, weekly, or on a daily basis. In addition, Rome IV criteria were used which are a set of diagnostic criteria and guidelines for functional gastrointestinal disorders. These criteria were published in 2016 and are an update to the previous Rome III criteria. The diagnostic criteria for functional constipation include experiencing two or more of the following symptoms for at least three months, with symptom onset at least six months prior to diagnosis: straining during more than 25% of defecations, lumpy or hard stools (BSFS 1-2) more than 25% of defecations, sensation of incomplete evacuation more than 25% of defecations, sensation of anorectal obstruction/blockage more than 25% of defecations, manual maneuvers to facilitate more than 25% of defecations, and fewer than three spontaneous bowel movements per week. Components of the questionnaire were adopted from the relevant literature assessing the clinical presentation of the disease and the associated risk factors [11]. The content of the questionnaire was reviewed by a consultant in surgery to assess the content validity of the questionnaire. Furthermore, face validity in the targeted community was assessed via piloting the questionnaire of a sample of 10 male and 10 female participants to investigate the clarity of the measurement tool and time needed to complete the questionnaire.

Data collection process

The questionnaire was transformed into an online format to enable distribution of the questionnaire in the relevant social media platforms. Identification and approaching of the participants were performed via advertising the questionnaire to the relevant population in the Jazan region. The study included adults who had undergone hemorrhoidectomy, were diagnosed with hemorrhoids, and experiencing related symptoms of hemorrhoids and constipation, or residing in Jazan. Individuals who did not meet these criteria, including minors, those who did not undergo hemorrhoidectomy, those without a diagnosis of hemorrhoids, those not complaining of symptoms, or those not residing in Jazan, were excluded from the study. The sample size for this research was determined using the Raosoft sample size calculator (Raosoft Inc., Seattle, WA, USA, raosoft.com), and 385 hemorrhoid patients were required to achieve a 95 percent confidence interval and a 5% margin of error assuming a prevalence of hemorrhoid was 50% as no previous studies were conducted in the region to estimate the prevalence of the disease. Selecting 50% as a targeted prevalence would provide the largest required sample given the provided confidence interval and margin of error. 

Data analysis 

Data set-up and analysis were performed using IBM SPSS Statistics for Windows, Version 25 (Released 2017; IBM Corp., Armonk, New York, United States). The descriptive analysis involved calculating frequencies and proportions of binary and categorical variables and estimating means, standard deviations, or medians, and interquartile range to summarize continuous data according to their distribution. The inferential statistics of the study was performed via the chi-squared test. The parameters used in the test were related to assessment of the frequency of hemorrhoid symptoms according to the measured sociodemographic and lifestyle characteristics of the participants. To enable performing the association, continuous variables, such as age, were converted to binary variables by dividing the sample using median as a cut-off point. Furthermore, categorical variables, such as clinical presentation of hemorrhoids, were converted into binary variables by grouping the sample into those who experience occurrence of six symptoms at least one or less than six symptoms per month and those who experience more than six symptoms per month. A p-value of less than 0.05 was presumed as a statistically significant value for the applied test. 

## Results

The total number of participants who were recruited in the current investigation was 361 participants, of whom 216 (59%) reported experiencing a minimum of one hemorrhoid symptom. Table 1 displays the demographic characteristics of the study’s participants. The majority of the samples were females (60%), Saudis (96%), and living in urban areas (59%) and had a university education (77.3%). More than half of the sample were married (57%) and were either employed, business owners, or students (68%). Finally, among the recruited participants, nearly one-third reported being ever tobacco smokers (29.4%), one quarter being Khat chewers (25%), and only seven participants (1.9%) reported being ever tobacco chewers. 

**Table 1:** Demographic characteristics of 361 adult participants from Jazan, Saudi Arabia

Variable	Frequency [proportion]
Gender	
Male	144 [39.9%]
Female	217 [60.1%]
Nationality	
Saudi	346 [95.8%]
Non-Saudi	15 [4.2%]
Place of residence	
Urban	214 [59.3%]
Rural	147 [40.7%]
Social status	
Single	123 [34.1%]
Married	205 [56.8%]
Divorced	23 [6.4%]
Widowed	10 [2.8%]
Educational level	
Able to read and write	5 [1.4%]
Elementary	5 [1.4%]
Intermediate	2 [0.6%]
Secondary	70 [19.4%]
University	279 [77.3%]
Job status	
Government employee	107 [29.6%]
Private sector employee	43 [11.9%]
Private business owner	6 [1.7%]
Student	89 [24.7%]
Housewife	41 [11.4%]
Retired	29 [8.0%]
Unemployed	43 [11.9%]
Khat chewing	
Ever chewer	89 [24.7%]
Never chewer	272[75.3%]
Smoking	
Ever smoker	106 [29.4%]
Never smoker	255 [70.6%]
Tobacco chewing	
Ever user	7 [1.9%]
Never user	352 [97.5%]

Table 2 displays the distribution of hemorrhoid risk factors and history of diagnosis and management of the disease among the participants. The most frequently reported risk factors are the lack of regular physical activity (83%), followed by sitting for long hours during office work (51%), and consuming food with saturated fat (50%). One hundred fifty-nine participants (44%) reported having a family history of hemorrhoids and a minority of the sample reported being diagnosed with a chronic disease. When the participants were asked about their defecation practices, 51% of the sample reported making an effort while defecating 25% of the time, 47% of the participants reported feeling their stool was stiffer than normal 25% of the time, and 40% of the sample reported suffering from constipation three times or more per week. Only 123 participants (34%) reported being diagnosed with hemorrhoids by a physician. When the participants were asked about treatment options for hemorrhoid symptoms, the majority (57%) reported using home remedies indicating preference of the participants to use home remedies rather than seeking medical care from a health professional.

**Table 2:** Hemorrhoid risk factors, history of diagnoses, and management of the disease among 361 participants from Jazan, Saudi Arabia

Variable	Frequency [proportion]
Does your work involve sitting for long office hours?	
Yes	185 [51.2%]
No	75 [20.8%]
I don’t work	101[28.0%]
Do you suffer from any of these chronic diseases?	
High blood pressure	3.4 [9.4%]
Diabetes	24 [6.7%]
Irritable bowel	22 [6.1%]
Respiratory diseases (asthma)	18 [5%]
Crohn's disease	4 [1.1%]
Ulcerative colitis	14 [3.9%]
Immune diseases	6 [1.7%]
Mental illnesses	12 [3.3%]
Do you exercise regularly?	
Yes	60 [16.6%]
No	301 [83.4%]
Does your diet contain a high amount of fiber or do your daily meals contain a high amount of fiber? (Mainly found in fruits, vegetables, whole grains, and legumes)?	
Mostly	36 [10%]
Once a day	62 [16.9%]
2-3 times a day	184 [51%]
Never	80 [22.2%]
Does your diet contain saturated meals?	
Mostly	181 [50.1%]
Once a day	76 [21.1%]
2-3 times a day	90 [24.9%]
Never	14 [3.9%]
Do you have chronic diarrhea?	
Yes	22 [6.1%]
No	339[93.9%]
How often do you suffer from constipation during the week?	
Less than three times per week	216 [59.8%]
Three times or more per week	145 [40.2%]
Do you feel like you’re making an effort while defecating?	
Less than 25%	177 [49%]
More than 25%	184 [51%]
Do you feel that the stool is stiffer or stiffer than normal?	
Less than 25%	190 [52.6%]
More than 25%	171 [47.4%]
Do you have a family history of hemorrhoids?	
Yes	159 [44%]
No	202[56%]
Have you been diagnosed by a doctor with hemorrhoids at a hospital?	
Yes	123 [34.1%]
No	238[65.9%]
Have you done any of the following treatments for hemorrhoids?	
Treatment with medicines	125 [34.9%]
Herbal remedy	75 [20.8%]
Home remedies	208 [57.6%]
Surgical intervention	29 [8.1%]

Table 3 displays the distribution of hemorrhoid symptoms among the participants. The most frequently reported symptom was experiencing a hemorrhoidal mass, followed by feeling itchy or uncomfortable around the anus. The majority of the samples never experienced symptoms related to bleeding or discharge during defecation. Assessment of factors associated with having a minimum of six hemorrhoid symptoms occurring per month indicates that being older than 33 years, being a male, being married, divorced, or widowed, being a khat chewer, or ever smoker, having a family history of hemorrhoids, sitting for prolonged hours during office work, having physical activity irregularity, low consumption of high diet fibers, having more frequent occurrence constipation, having stiffer stool, and making effort while defecation were associated with higher occurrence of hemorrhoid symptoms with statistically significant values (<0.05) (Table 4). 

**Table 3:** Distribution of hemorrhoid symptom occurrence among a sample of 361 participants from Jazan, Saudi Arabia

Symptoms	Never	Less than once monthly	Less than once weekly	1-6 times per week	Every day
How frequent are the appearance of the hemorrhoidal masses (swelling)?	133 [36.8%]	63 [17.5%]	29 [8%]	58 [16.1%]	78 [21.6%]
How often do you feel pain from the hemorrhoids?	204 [56.5%]	42 [11.6%]	64 [17.7%]	51 [14.1%]	0
How often do you feel itchy or uncomfortable in the anus?	195 [54%]	53 [14.7%]	55 [15.2%]	58 [16.1%]	0
How many times do you bleed while defecation?	260 [72%]	22 [6.1%]	37 [10.2%]	42 [11.6%]	0
How often does your underwear get dirty as a result of discharge from the anus?	259 [71.7%]	25 [6.9%]	33 [9.1%]	44 [12.2%]	0
How often do you feel swelling or sagging hemorrhoids?	217 [60.1%]	28 [7.8%]	48 [13.3%]	68 [18.8%]	0

**Table 4:** Association between demographic factors and frequency of hemorrhoid symptoms among 216 participants from Jazan, Saudi Arabia with a minimum of one hemorrhoid symptom occurring per month

Variables	Symptoms			
	1 or less than 6 symptoms per month	6 symptoms or more per month	Total	P value
Age				
33 years or less	70 [65.4%]	37 [34.6%]	107 [100%]	<0.001
More than 33 years	42[38.5%]	67[61.5%]	109[100%]	
Gender				
Male	28[28.3%]	71[71.7%]	99[100%]	<0.001
Female	84[71.8]	33[28.2%]	117[100%]	
Education				
Less than university education	24[57.1%]	18[42.9%]	42[100%]	0.494
University education	88[50.6%]	86[49.4%]	174 [100%]	
place of residence				
Urban	62[50.4%]	61[49.6%]	123[100%]	0.681
Rural	50[53.8%]	43[46.2%]	93[100%]	
Social status				
Single	44[69.8%]	19[30.2%]	63[100%]	<0.001
Married	64[50%]	64[50%]	128[100%]	
Divorced	3[18.8%]	13[81.3%]	16[100%]	
Widowed	1[11.1%]	8[88.9%]	9[100%]	
Khat				
Ever chewer	9[12.2%]	65[87.8%]	74[100%]	<0.001
Never chewer	103[72.5%]	39[27.5%]	142[100%]	
Smoking				
Ever smoker	16[19.3%]	67[80.7%]	83[100%]	<0.001
Never smoker	96[72.2%]	37[27.8%]	133[100%]	
Shamma				
Ever use	1[14.3%]	6[85.7%]	7[100%]	0.056
Never use	111[53.6%]	96[46.4%]	207[100%]	
Do you have a family history of hemorrhoids?				
Yes	46[36.2%]	81[63.8%]	127[100%]	<0.001
No	66[74.2%]	23 [25.8%]	89 [100%]	
Does your work involve sitting for long office hours?				
Yes	50[39.4%]	77[60.6%]	127[100%]	<0.001
No	28[68.3%]	13[31.7%]	41[100%]	
I don’t work	34[70.8%]	14[29.2%]	48[100%]	
Do you exercise regularly?				
Yes	20[74.1%]	7[25.9%]	27[100%]	0.022
No	92[48.7%]	97[51.3%]	189[100%]	
Does your diet contain a high amount of fiber or do your daily meals contain a high amount of fiber? (Mainly found in fruits, vegetables, whole grains and legumes)				
Mostly	16[84.2%]	3[15.8%]	19[100%]	<0.001
Once a day	19[63.3%]	11[36.7%]	30[100%]	
2-3 times a day	56[53.3%]	49[46.7%]	105[100%]	
Never	21[33.9%]	41[66.1%]	62[100%]	
Does your diet contain saturated meals?				
Mostly	46[36.2%]	81[63.8%]	127[100%]	<0.001
Once a day	24[68.6%]	11[31.4%]	35[100%]	
2-3 times a day	37[75.5%]	12[24.5%]	49[100%]	
Never	5[100%]	0[0.0%]	5[100%]	
Do you have chronic diarrhea?				
Yes	8[53.3%]	7[46.7%]	15[100%]	1.000
No	104[51.7%]	97[48.3%]	201[100%]	
Do you have constipation?				
Yes	60[41.7%]	84[58.3%]	144[100%]	<0.001
No	52[72.2%]	20[27.8%]	72[100%]	
How often do you suffer from constipation during the week?				
Less than 3 times per week	77[74%]	27[26%]	104[100%]	<0.001
Three times or more per week	35[31.3%]	77[68.8%]	112[100%]	
Do you feel like you’re making an effort while defecation?				
Less than 25%	58[82.9%]	12[17.1%]	70[100%]	<0.001
More than 25%	54[37%]	92[63%]	146[100%]	
After defecation do you feel uncomfortable?				
Less than 25%	75[51.7%]	70[48.3%]	145[100%]	1.000
More than 25%	37[52.1%]	34[47.9%]	71[100%]	
Do you feel that the stool is stiffer than normal?				
Less than 25%	63[72.4%]	24[27.6%]	87[100%]	<0.001
More than 25%	49[38%]	80[62%]	129[100%]	

## Discussion

This investigation was a cross-sectional study aiming to assess the prevalence of hemorrhoid symptoms and risk factors among adult subjects residing in Jazan. More than half of the participants experienced a minimum of one hemorrhoid symptom and a high prevalence of certain hemorrhoid risk factors such as limited physical activity and occupational risk factors. Nonetheless, only one-third of the sample reported being diagnosed with hemorrhoids by a physician. Furthermore, more than half of the sample utilized home remedies, and one-fifth reported the use of herbal medications. This healthcare-seeking behavior may partially explain patient's preference indicating that those participants who experience hemorrhoid symptoms may prefer not to seek advice from a healthcare professional at earlier stages of the disease. 

The findings of the current study can be compared to similar local and international studies. The findings of the current study indicate high prevalence of lifestyle risk factors of hemorrhoids among the population in Jazan and frequent reporting of symptoms associated with hemorrhoids. However, it must be noted that studies that assessed the prevalence of hemorrhoids in Saudi Arabia are limited. A study conducted in the Western region of Saudi Arabia assessed the prevalence of post-hemorrhoidectomy complications which indicated that patients who lived in mountain areas in the city of TAif were likely to experience complications and recurrence of hemorrhoid symptoms in comparison to those who are living costal and non-mountain areas in the city of Jeddah, where this variation was attributed to the difference in altitude [12]. Though our investigation did not perform a similar comparison, the region of Jazan has similar geographical variation. Forty percent of our sample reported suffering from constipation three or more times per week and it was significantly associated with the reporting hemorrhoid symptoms. This indicates the importance of constipation as a prevalent risk factor for hemorrhoids in the region of Jazan. A study that assessed awareness of 778 participants from the western region of Saudi Arabia about constipation indicated that being younger, having lower education, and not having experienced constipation were associated with lower awareness of constipation and its associated complications including hemorrhoids [13]. Lifestyle factors associated with hemorrhoids such as limited physical activity, sitting for long hours during work, smoking, khat chewing, and consumption of diets with low fiber contents and high saturated fat were identified in our sample. This is similar to the findings of other international studies indicating high BMI [14,15] and limited physical activity [16], indicating the importance of addressing lifestyle factors in health education and management of hemorrhoid patients [17].

Our investigation suggests that although some adults may experience symptoms associated with hemorrhoids, the presence of hemorrhoid symptoms may not motivate the patient to seek advice from a healthcare professional. This notion is supported by an Austrian study that involved 976 adult participants from the general population who underwent colorectal cancer screening, which included performing colonoscopy and detection of hemorrhoids. It was concluded that nearly 39% of participants suffered from hemorrhoids with variable degrees and reported that a considerable proportion of patients who suffer hemorrhoidal symptoms do not complain about the symptoms [14]. Similarly, in a Chinese study that involved 306 patients diagnosed with hemorrhoids, it was noted that middle-aged patients are likely to experience a delay in seeking healthcare when experiencing hemorrhoidal symptoms [18]. Hemorrhoids are a common problem in the region, and they can significantly impact patients' quality of life. Hemorrhoids can have a considerable negative influence on the overall population regarding health and socioeconomics. To prevent developing hemorrhoids and its problems, it is also essential that the general public is aware of healthy lifestyle choices and takes into account all available treatment options. By identifying the factors contributing to the development of hemorrhoids, it is possible to develop effective strategies to prevent and manage this condition. 

Limitation

Our study has multiple areas of strengths and weaknesses. Our investigation was able to reach a general population sample to assess the prevalence associated with hemorrhoids and their risk factors via utilizing an online approach. Nonetheless, a possibility of selection bias is present as subjects with limited access to the internet are less likely to participate. However, it is possible to argue that use of an anonymous online tool is likely to pose less embarrassment when asking about hemorrhoid symptoms in comparison to face-to-face interviews. 

Recommendation

The findings of the current study suggest a need to increase the awareness of the public about hemorrhoid risk factors and educate the Jazan population about the importance of seeking healthcare at an early stage of the disease and encouraging healthy habits by emphasizing the importance of adopting a healthy lifestyle to prevent hemorrhoids by promoting regular exercise, a balanced diet rich in fiber, and adequate hydration. Furthermore, the study suggests engaging local healthcare providers, collaborating with local healthcare providers to ensure accurate and up-to-date information is disseminated, requesting them to display educational materials at their clinics, and considering partnering with healthcare professionals for interactive sessions or Q&A sessions related to hemorrhoids.

## Conclusions

The current study identified a high prevalence of hemorrhoid symptoms among the adult population in the Jazan region of Saudi Arabia. Similarly, high prevalence of hemorrhoid risk factors was identified in the sample including low physical activity, long sitting periods during working hours, and consumption of food items with low fiber contents and high saturated fat. Additionally, high prevalence of constipation and a family history of hemorrhoids are identified. Nearly one-third were diagnosed with hemorrhoids. The participants indicated a preference to use whom remedies rather than visiting a healthcare provider.
